# BME-free primary patient-specific organoids obtained with a one-day mimicking method to replicate the corresponding tumor for personalized treatment options

**DOI:** 10.3389/fonc.2023.1239957

**Published:** 2023-12-15

**Authors:** Yan Zhu, Zhechun Ding, Yini Wang, Qing Wu, Dongmei Chen, Luanhong Wang, Yuancheng Li, Yao Yao, Jiman Huang, Yun Li, Xiaojing Wang, Yanchun Lin, Tian Guan, Haoyu Zeng, Congzhu Li

**Affiliations:** ^1^ Department of Gynecological Oncology, Tumor Hospital Affiliated to Medical College of Shantou University, Shantou, China; ^2^ Department of Cancer Research, Guangdong Procapzoom Biosciences, Inc., Guangzhou, Guangdong, China

**Keywords:** patient specific, organoid, tumor, personalized treatment, gynecologic cancer

## Abstract

**Introduction:**

In cancer treatment, every minute counts. Due to the unpredictable behavior of cancer cells caused by continuous mutations, each cancer patient has a unique situation and may or may not respond to a specific drug or treatment. The process of finding an effective therapy can be time-consuming, but cancer patients do not have the luxury of time for trial and error. Therefore, a novel technology to fast generate a patient relevant organoid for the therapies selecting is urgently needed.

**Methods:**

Utilizing the new organoid technology by specially dissolving the mesenchyme in tumor tissues acquired from cancer patients, we realized the work of creating patient-specific organoids (PSO) within one day.

**Results:**

PSO properties reflect those of its respective original in vivo tumor tissue and can be utilized to perform various in vitro drug sensitivity tests to identify the most effective clinical treatment for patients. Additionally, PSO can aid in assessing the efficacy of immune cell therapies.

**Discussion:**

Organoid technology has advanced significantly in recent years. However, current cancer organoid methods involve creating 3D tumor tissue from 2D cancer cells or cell clusters, primarily for cancer research purposes aimed at investigating related molecular and cellular mechanisms of tumor development. These methods are research-driven, not tailored towards clinical applications, and cannot provide personalized information for individual patients. PSO filled the gap of clinic-driven and time-saving method for the personalized therapies selecting to the cancer patients.

## Introduction

Organoid culture systems have rapidly emerged as promising tools in various research studies on human diseases and their phenotypic and molecular aspects (1). Organoids have been utilized as models for normal human organs, including the bladder (2), breast (3), colon (4), liver (5, 6), lung (7), ovarian (8), pancreas (9), prostate (10), and stomach (11). Similarly, cancer organoids of these human tissues have been created using cell lines, cells differentiated from stem cells, and patient-derived tissues, including bladder (12), breast (3), colorectal (13), liver (14), lung (15), ovarian (16), pancreatic (9), prostate (17), and gastrointestinal cancers (18). Traditional cancer organoids have the advantage of being capable of large-scale culture and useful in contributing tumor characteristics to bio- and gene-banks. Aside from exploring the underlying molecular and cellular mechanisms of tumor development (1, 19), cancer organoids have been effectively utilized in drug screening, although they are passaged rather than primary organoid cultures (12, 13, 15–18). As a result, the screening results are only applicable to organoids and not individual patients. As *in vitro* passaging leads to mutations, the properties of passaged organoids are no longer equivalent to those of their original tumor tissues (20).

Each cancer patient is unique, and their tumor may or may not respond to specific drugs or treatment regimens. The current trial and error approach is not an option for cancer patients, as it may lead to a loss of hope and life. Due to limited time, cancer patients need efficient treatment, which highlights an unmet need for the development of an alternative approach to conduct drug screening for individuals, rather than relying solely on a trial-and-error method. However, one of the biggest challenges is maintaining the individual tumor properties for each patient throughout the screening process.

Our primary objective is to preserve the unique tumor features of individual patients for personalized ex vivo drug testing, ensuring the selection of the most effective drug for their specific tumor.

While we have previously generated and banked conventional organoids derived from patients, which have proven advantageous for cancer research and high-throughput drug candidate screening due to their abundance and ease of culturing, there is a notable limitation when it comes to using traditional organoids for individual patients. The well-known phenomenon of tumor cell variability during *in vitro* culture raises doubts regarding whether a traditional organoid is truly representative of the tumor in the patient’s body.

To address this limitation, we altered our strategy to minimize the timeframe required for organoid preparation, aiming to maintain their original features to the greatest extent possible. We have developed a novel organoid technology that utilizes a specialized culture medium to create patient-specific organoids (PSO) within one day using tumor tissue obtained from cancer patients. Our method features a very short one-day preparation time, which differs from current cancer organoid technologies that involve digesting tumor tissue into three to five cell clusters and regenerating them into organoids over several days (21). These PSOs serve as a valuable resource for small-scale therapeutic selection and provide valuable recommendations for both doctors and patients. It is worth mentioning that similar ideas have been explored by others in studies such as Schuth, S., et al., 2022 (22) and Tsai, S., et al., 2018 (23), where primary organoids were employed for personalized therapy selection.

Additionally, unlike other methods, our PSO approach does not require extracellular substances, making our PSOs ready for use in a single day while retaining the original tumor characteristics. Consequently, PSOs can be used to conduct *in vitro* drug sensitivity testing, providing a relative efficacy ranking of available treatment options for the particular patient whose tumor is the origin of the PSO, without subjecting patients to the current “trial and error” approach, thereby saving their time and life. Furthermore, our method enables direct tissue-level evaluation of the effects of immune cells that cannot penetrate the gel matrix used in other methods by attaching PSOs to the bottom of a 96-well plate without Matrigel (24).

## Materials and methods

### PSO preparation

**Figure 1:**
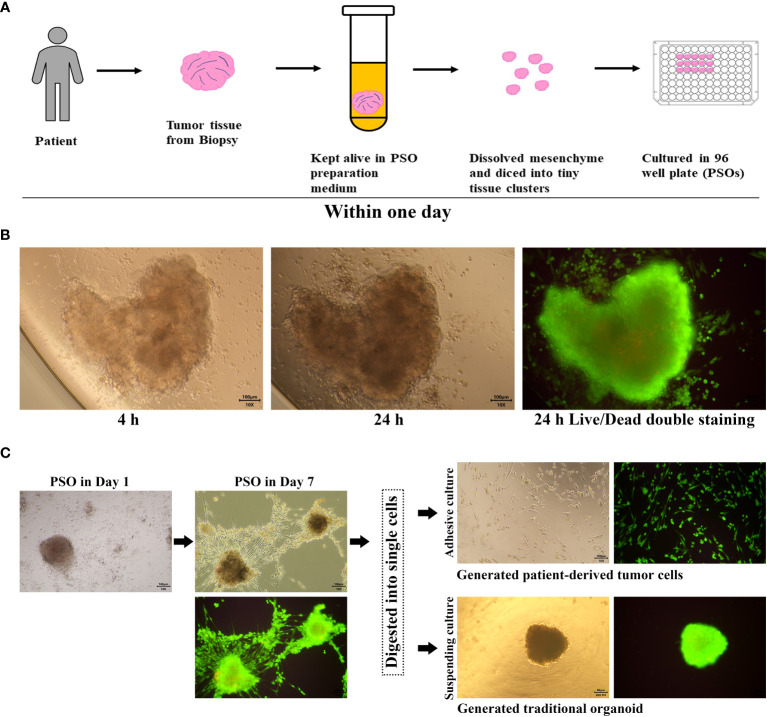
PSO procedure and proliferation potential. **(A)** Our process for generating PSO from tumor tissue involves only one day. **(B)** Within this one-day period, we were successful in isolating tiny tissue clusters from the tumor (4 hours) and generating PSO (24 hours), which had a survival rate of over 90%, as evidenced by green staining. **(C)** PSO can continue to grow in a BME-Free environment for at least one week and can transform into tumor cells or traditional organoids following the passaging process.

Patient tumor samples were obtained via biopsy from the Tumor Hospital, Medical College of Shantou University with patients’ acknowledgment. Fresh tumor samples were kept alive using PSO preparation kit (#CCMP20, Procapzoom, Guangdong, China) and transferred into GMP level laboratory for further process per manufacture’s specification. Briefly, samples were washed 4-6 times in washing buffer, cut into 1 mm^3^ size tissues, and dissolved mesenchyme with dissolving buffer A (hyaluronidase as main component) for 60 min at 37°C. After dissolving, the tissues were diced to small clusters below 1 mm^3^ size, filtrated and those with diameters within 50 μm to 500 μm were retained. The selected clusters were soaked in buffer B (containing hyaluronidase, DNase, cellulases, collagenase I, collagenase II and collagenase IV), in which we improved the ratio of hyaluronidase: DNase: collagenase as 2: 1: 2.5, for 30 min to further dissolve fibrous tissue and hyaluronic acid. Then the dissolved clusters were cultured in an incubator at 37°C with 5% CO_2_ using a proprietary medium from the kit for 1 day to generate PSOs (**Figures 1A, B**). Finally, PSOs were attached in a Gelatin (ST1339, Beyotime, China) coated with 96 well plate as a 3D formation and ready for therapy selecting (**Figures 1A, B**). For the traditional organoids re-generated from PSO, the method was performed according to the previous study (21). Briefly, the PSO was digested with 0.25% trypsin (#25200056, Gibco, USA) for 2 min to single cells, then 3-5 single cells were gathered and embedded in the BME (3533-005-02, R&D Systems, USA) for about 2 weeks. The spheroids with the diameters during 200 μm to 500 μm were selected for further investigation of comparison with the PSOs.

### Immunohistochemistry and H&E staining

For immunohistochemistry (IHC), tumor tissues and PSOs were fixed with a 4% paraformaldehyde solution and embedded in agarose. The samples embedded in agarose were dehydrated, paraffin-embedded, and sliced into 4-μm sections. After deparaffinization and rehydration, the sections underwent a 10-minute treatment with 3% hydrogen peroxide to expand endogenous peroxidase activity. Subsequently, the tissue sections were autoclaved at 95°C in citrate buffer (pH 6.0) for 5 minutes for antigen retrieval. Then the sections were blocked with 5% BSA and incubated with the antibody MSLN (ab196235, Abcam, USA), MUC1 (ab70475, Abcam, USA), and CD276 (ab227670, Abcam, USA) primary antibodies overnight at 4°C. The GTVision antibody complex (anti-mouse/rabbit) method was used for color development, and the chromogen substrate was the 3,30-diaminobenzidine from the GTvision I Detection System kit (Gene Tech Co, Ltd, Shanghai, China). For Hematoxylin and Eosin (H&E) staining, tumor tissues and PSOs were fixed with a 4% paraformaldehyde solution, dehydrated, paraffin-embedded, microtome sectioned, and stained with the Hematoxylin and Eosin kit (ab245880, Abcam, USA) according to the manufacturer’s specifications.

### Drugs screening and live cells measuring of PSO

PSOs were cultured in 96-well plates, with 2-3 PSOs per well. Eight wells constituted each test group, and each test group was treated with various chemotherapeutic drugs or targeted drugs, such as paclitaxel (A71384, Innochem, China), sirolimus (53123-88-9, MedChemExpress, USA), oxaliplatin (A27295, Innochem, China) and capecitabine (C124969, Aladdin, China). The drugs were administered at the respective clinical-relevant concentrations, as indicated in the figure legends, for a period of 5-7 days. Subsequently, Live/Dead cell double staining kit (KGAF001, KeygenBiotech, China) was used to stain the PSOs, per the manufacturer’s specifications. After staining, the PSOs were visualized using a fluorescence microscope (BDS400, Cnoptec, China) with 500-550nm excitation light. The green and red fluorescence emissions were isolated and analyzed separately. The mean fluorescence intensities of each group were then quantified using ImageJ software (version v1.53h, NIH).

### Co-culturing of PSOs and immune cells

PSOs were prepared in the 96-well plates for 1-2 PSOs in each well. The volumes of PSOs were uniformed as better and the diameters under the size of 500 μm. Before co-culturing, the live cell staining (green fluorescence) regent of the Live/Dead cell double staining kit was added into the wells for 40 minutes, to mark all the live cells in PSOs with green fluorescence, then had been washed out with DPBS. Subsequently, 1×10^5^ numbers of immune cells were added into each well with 750 uL immunocyte culturing medium. The wells containing PSOs should have another 750 uL PSO culturing medium. After 8 hours or longer co-culturing in an incubator at 37°C with 5% CO_2_, the PSOs were observed and photographed under fluorescence microscope (BDS400, Cnoptec, China) with 500-550nm excitation light.

### Quantitative real-time PCR

Total RNA from the tumor tissues, PSOs, and traditional organoids were extracted using the Total RNA Extraction Kit (#19221ES50, Yeasen Biotech, China), following the manufacturer’s protocol. qPCR was performed using the Accurate 96 Real-Time PCR machine (DLAB Scientific, Beijing, China) with the Hieff® qPCR SYBR Green Master kit (#11203ES08, Yeasen Biotech, China), as per the manufacturer’s instructions. All samples were set triplications (n≥3). The expression levels of the measured genes were normalized to the internal control and analyzed using the 2−ΔΔCt method. Primers of relevant genes have been listed in **Table S1**.

### Flow cytometry

After culturing, PSOs were digested with 0.25% trypsin (#25200056, Gibco, USA) for 2 min to single cells. The tumor tissues were firstly pieced and digested according the previous study by *Kar, R., et al., 2017* (25), and then following an additional step of digesting with 0.25% trypsin for 15 min, resuspending and filtering to obtained single cells. Then the cells were measured using a flow cytometer (Sparrow, Celula, USA) and analyzed by the software FlowJo (version 10, FlowJo Company, USA). The antibodies for biomarkers determination were using anti-mesothelin antibody (ab252136, Abcam, USA), anti-human CD276 (B7-H3) antibody (351005, Biolegend, USA) and anti-human CD227 (MUC-1) Antibody (355603, Biolegend, USA).

### Cell viability assays

SKOV-3 cells or PSOs were seeded into a 96-well plate for 2×10^5^ cells or 5 PSOs per well. After being treated with different drugs and continuously cultured for 48 hours, the cells or PSOs were washed twice with DPBS and replaced with fresh medium. The viability of the cells or PSOs were then analyzed by performing CCK-8 assays (C0039, Beyotime Biotechnology, China). Briefly, the CCK-8 solution was added to each well for 10 µL per well and then the 96-well plate were incubated at 37°C for two hours. PSOs needed to be digested with 0.25% trypsin for 2 min and resuspended into single cells before adding CCK-8 solution, in order to avoid the interference of light obstruction. Finally, the microplate reader (PT-3502B, Potenov, Beijing, China) was used to determine absorbance at the OD = 450 nm.

### Wound healing assay

The SKOV-3 cell line was seeded in to 12-well plates for 1 × 10^5^ cells per well. The cells were cultured in the incubator at 37° with 5% CO_2_ and generated to the confluence at about 90%. Then the wells were scratched by sterile pipette tip at the bottom and washed twice with DPBS to remove detached cells. The cells were subsequently cultured in medium for 24 hours. The scratches were monitored at the 0^th^, 10^th^ and 24^th^ hour.

### Statistical analysis

All the quantification data were calculated and graphed with software Prism GraphPad 8 (version v8.3.0, GraphPad Software Inc). The statistical analyses were performed using One-way ANOVA or Two-way ANOVA to compare differences between mean of each treated group and mean of control group for single variate or multiple variables. The difference with the p < 0.05 was considered as statistically significant difference, indicated by “*”, and P < 0.01 was indicated by “**”.

## Results

The procedures for preparing PSO were described in the Materials and Methods section, as shown in **Figure 1A**. The key aspect of the preparation involved dissolving mesenchymal components such as fibrin and collagen from the tumor tissues by exposing them to a dissolving buffer for more than an hour at a temperature of 37°C.

Additionally, we have improved the ratio of reagents as described in the method. These innovations have enabled us to obtain tiny primary tissues that are more structurally complete and composed of live tumor cells, rather than regenerated ones.

Tumor tissues were procured from clinical patients and kept in a specially-formulated medium until tiny tissue clusters could be successfully isolated. Within a day, these tiny clusters grew into PSOs in a 96-well plate and were then ready for subsequent assays, as depicted in **Figure 1A**. Under BME-Free conditions, a single tiny tissue cluster was able to grow into a PSO within a day, as long as it had its own cell binding structure, as shown in **Figure 1B**. Live/Dead cell double staining revealed that the PSO was generated with about 90% living cells, demonstrating its high viability. Moreover, we observed that PSOs could continue to proliferate for at least a week, as presented in **Figure 1C**. When passaged with cell digestion, these PSOs could also be transferred into traditional organoids with suspending culturing or primary cells with adhesive culturing, as illustrated in **Figure 1C**. To investigate the composition of the PSO body, we conducted immunohistochemistry to detect Ki67-positive cells. Additionally, we examined the expression of CD90, CD105, and CD73 to determine the ratio of fibroblasts among the migrated cells originating from the PSO body. Our findings revealed that the majority of migrated cells were indeed fibroblasts (**Figure S1A**), while the Ki67-positive cells were confined to the cancer nests within the main body of the PSO (**Figure S1B**). This observation suggests that PSOs have the capacity to protect themselves against senescence. However, it indicated that passaged and regenerated organoids from dissociated cells may carry the risk of altered fibroblast proportions in relation to their corresponding tumors.

**Figure 2:**
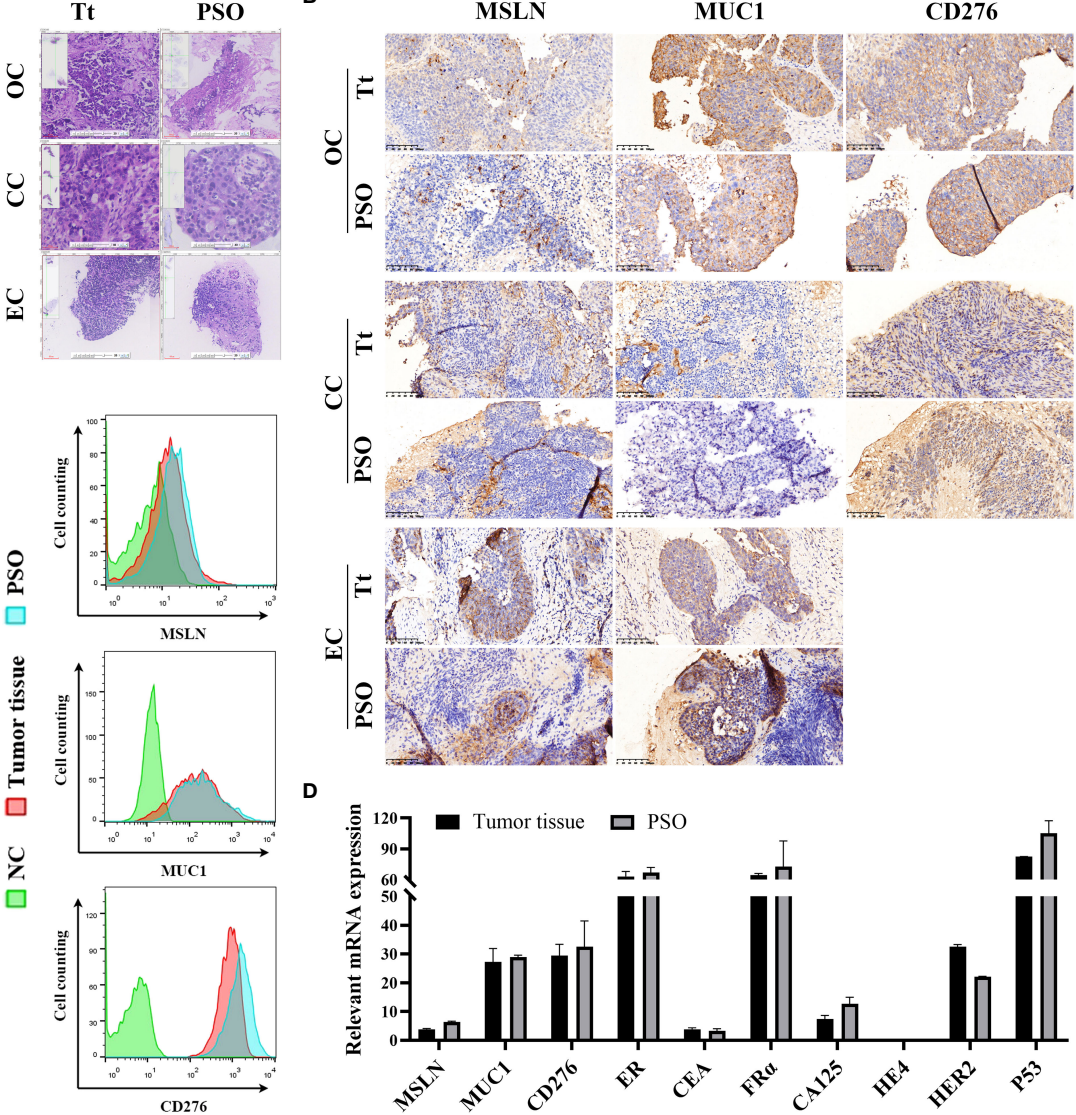
Similar properties between PSO and tumor tissue. **(A, B)** Our study utilized H&E staining **(A)** and IHC staining **(B)** to compare PSOs obtained from different gynecologic cancers to their corresponding tumor tissues. **(C)** We also conducted cell counting to determine the number of MSLN, MUC1, and CD276 positive cells in the PSO from ovarian cancer and the corresponding tumor tissue. Negative controls were cells that lacked relevant protein expression. **(D)** In addition, we analyzed relevant mRNA expression levels in the PSO obtained from ovarian cancer and the corresponding tumor tissue. Abbreviations used in this study include Tt (tumor tissue), OC (ovarian cancer), CC (cervical cancer), EC (endometrial cancer), and NC (negative control).

We have successfully developed a PSO model to replicate several types of gynecological cancers, specifically ovarian cancer (OC), cervical cancer (CC), and endometrial cancer (EC). The H&E staining results demonstrated that the PSOs of OC, CC, and EC exhibited nearly identical structures to the corresponding tumor tissues, as illustrated in **Figure 2A**. We also detected relevant surface biomarkers of these gynecological cancers, such as MSLN, MUC1, and CD276, in the PSOs and respective tumor tissues obtained from the same clinical samples. The expression patterns of these biomarkers in PSOs were consistent with those in tumor tissues, as presented in **Figure 2B**. In detail, the OC tumor tissue and PSO showed MSLN expression on some cells, as well as positive expression of MUC1 and CD276. The CC tumor tissue and PSO exhibited negative expression of MSLN and MUC1 in the parenchymal region and positive expression of CD276. The EC tumor tissue and PSO expressed MSLN and MUC1 positively in the parenchymal region, as shown in **Figure 2B**. Furthermore, flow cytometry results indicated that the ratio of positive cells expressing these biomarkers in the digested OC tumor tissue and corresponding PSO were similar, as shown in **Figure 2C**. Additionally, the mRNA expression level of several tumor-relevant genes in the OC tumor tissue and corresponding PSO exhibited similar trends, as presented in **Figure 2D**.

**Figure 3:**
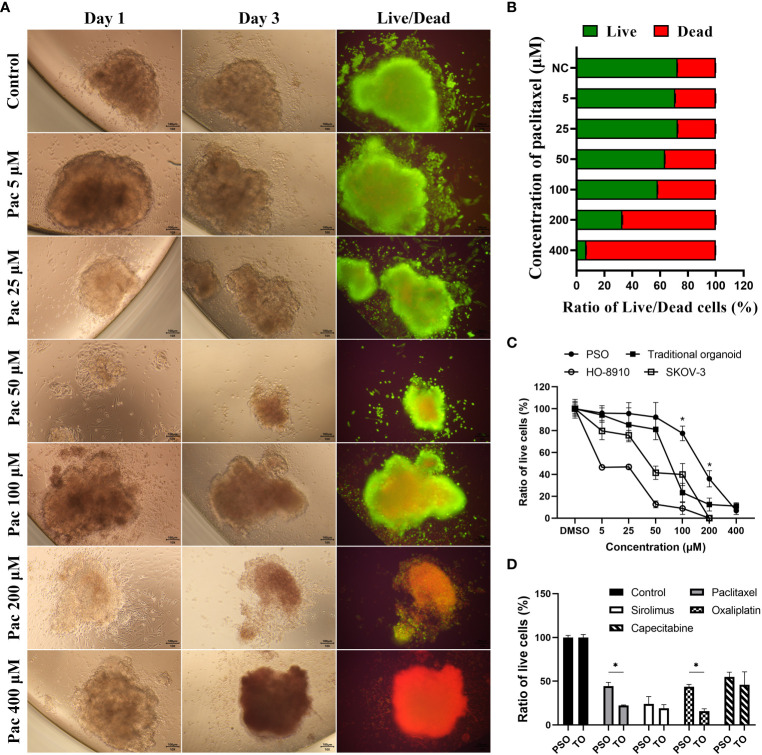
Drug treatment and screening test in PSOs. **(A, B)** To evaluate the efficacy of paclitaxel treatment on PSOs, we conducted fluorescent staining of live/dead cells **(A)** and obtained quantification results **(B)** following a paclitaxel gradient treatment. **(C)** We further compared the ratio of live cells in PSOs, traditional organoids, cell line HO-8910, and cell line SKOV-3 following paclitaxel gradient treatment for 24 hours. The ratio of live cells in each group was normalized to the DMSO control group (set as 100%). **(D)** We also evaluated the ratio of live cells in PSOs and traditional organoids following treatment with different drugs for 7 days. The results were normalized to the control group (DMSO) and presented as a percentage. Abbreviations used in this study include Pac (paclitaxel), TO (traditional organoid). “*”, statistically significant difference with the value of p < 0.05.

We assessed the viability of PSO using fluorescent Live/Dead cell staining. The results indicated that PSO could accurately reflect its viability under varying concentrations of paclitaxel treatments. As the paclitaxel concentration increased from 5 μM to 400 μM, the ratio of dead cells (red fluorescence) became higher, while the ratio of live cells (green fluorescence) decreased, as depicted in **Figure 3A**. The quantification data of the fluorescent Live/Dead cell staining are presented in **Figure 3B**.

Compared to traditional organoids and cell lines, PSO demonstrated higher drug resistance to paclitaxel. When subjected to the same concentration gradient of paclitaxel treatment, cell line HO-8910 demonstrated 50% mortality at 5 μM of paclitaxel, cell line SKOV-3 at 50 μM, and traditional organoids at approximately 75 μM, while PSO exhibited 50% mortality at nearly 200 μM, as shown in **Figure 3C**. Interestingly, PSO and the traditional organoid derived from the same sample exhibited different sensitivities to different chemotherapy agents, as illustrated in **Figure 3D**. Among the tested chemotherapy agents, PSO demonstrated higher drug resistance than traditional organoids under paclitaxel or oxaliplatin treatment, as depicted in **Figure 3D**.

**Figure 4:**
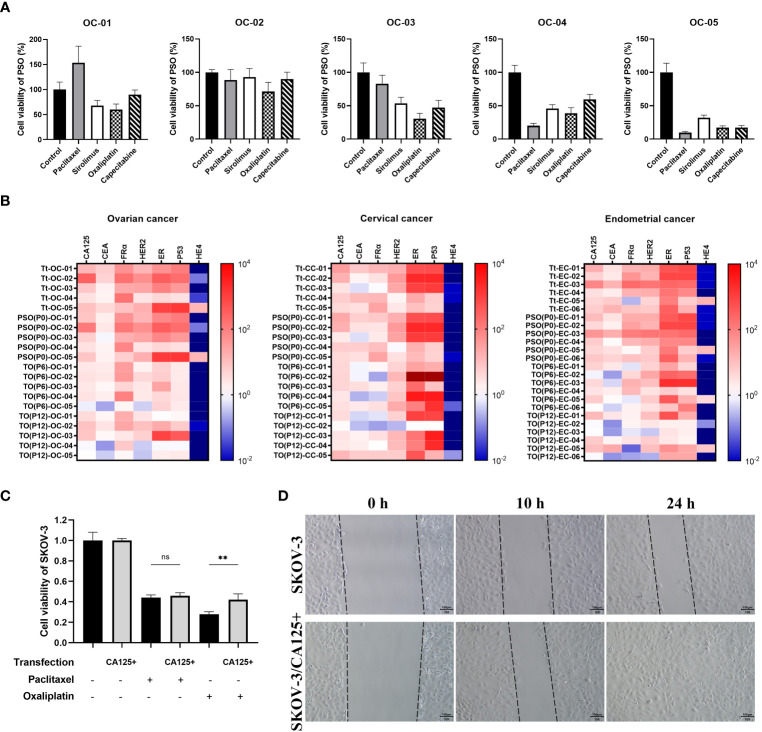
The potential of PSO for pre-clinical drug screening or future investigation. **(A)** To mimic pre-clinical drug screening, we used PSOs obtained from different ovarian cancer patients and treated them with paclitaxel (5μM), sirolimus (0.5μM) oxaliplatin (10μM) and capecitabine (1μM) for 7 days. **(B)** We also analyzed the relevant mRNA expression patterns of the tumor tissue, PSO, and traditional organoid at P6 or P12 from each of the patients with ovarian cancer (5 donors), cervical cancer (5 donors), or endometrial cancer (6 donors). **(C)** In addition, we evaluated the cell viability of SKOV-3 with or without CA125 overexpression after treatment with paclitaxel or oxaliplatin. The results were normalized to the control group, which was assigned a value of 1.0. **(D)** Lastly, we conducted a wound healing test of SKOV-3 with or without CA125 overexpression, and recorded the snap point at the 0^th^, 10^th^, and 24^th^ hour. Abbreviations used in this study include Tt (tumor tissue), TO (traditional organoid), OC (ovarian cancer), CC (cervical cancer), EC (endometrial cancer), and SKOV-3/CA125+ (SKOV-3 with CA125 overexpression). “**”, statistically significant difference with the value of p < 0.01. “ns”, statistically unsignificant difference.

We collected various tumor tissues from clinical patients with OC, CC, or EC and generated corresponding PSOs for different drug treatments to mimic clinical drug screening. The detailed clinical information of those patients could be checked in **Table S1**. As shown in **Figure 4A**, the cell viability of PSO after treatments varied widely between individual samples, even within the same cancer type. For example, PSO from sample OC-01 exhibited drug resistance to paclitaxel, leading to increased cell viability of up to 153%, while being most sensitive to oxaliplatin, which decreased cell viability to 60%. In comparison, PSO OC-02 displayed drug resistance to nearly all tested drugs, whereas PSO OC-05 was sensitive to all drugs tested and most sensitive to paclitaxel, which decreased cell viability to 9.6%. Other PSOs exhibited different trends in cell viability following drug treatments (**Figure 4A**).

To assess the suitability of PSOs as a drug screening model, we compared the relevant gene expression in the tumor tissues, PSOs, and traditional organoids. The results showed that PSOs of OC had a similar mRNA expression pattern to the corresponding tumor tissues for several clinically detected genes, including CA125, CEA, FRα, HER2, ER, P53, and HE4, as demonstrated in **Figure 4B**. However, when PSOs were generated to higher passages, as in traditional organoids, the mRNA expression pattern changed for some of these relevant genes, such as CA125, HER2, ER, and P53, while maintaining similarities in CEA, FRα, and HE4 (**Figure 4B**). The samples from CC and EC also displayed similar trends of expression changes in these relevant genes (**Figure 4B**).

Based on the drug resistance results and mRNA expression pattern, it was found that PSO OC-02 exhibited a higher level of drug resistance and the highest mRNA expression levels of CA125 and FRα as compared to other PSOs. It was also observed that PSOs as a whole demonstrated greater drug resistance than traditional organoids and showed higher average levels of CA125 and HER2 expression than traditional organoids.

With this in mind, further investigation was carried out with a focus on CA125, which was overexpressed in the SKOV-3 cell line. Analysis of the drug treatment results indicated that SKOV-3 cells with CA125 overexpression exhibited a higher level of resistance against oxaliplatin as shown in **Figure 4C**. In addition, the wound healing test results displayed higher tumor cell migration and regeneration ability in SKOV-3 cells with CA125 overexpression as compared to others, as demonstrated in **Figure 4D**. The similar trends could also be observed in the cell line HO8910 and patient primary tumor cells (**Figure S2**).

In recent times, immunocyte therapy has emerged as a newly explored method of clinical treatment. Besides drug screening, a further aim was to determine whether PSOs could serve as an appropriate model for screening immunocyte therapies. As compared to traditional organoids, which require embedding in BME or BME-like gels, PSOs are able to maintain their 3D structure in wells or dishes without the need for BME embedding.

**Figure 5:**
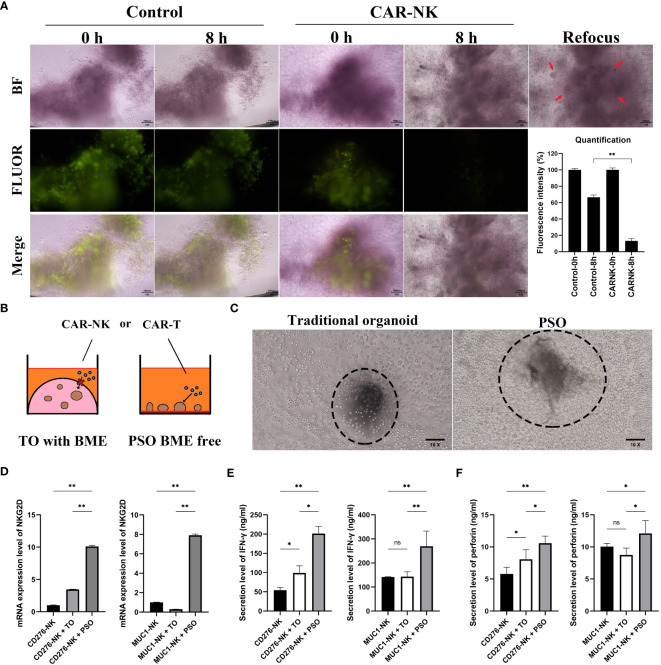
The potential of PSO for cell therapies testing. **(A)** To evaluate the efficacy of PSOs in cell therapies, we conducted fluorescent staining (green) of live cells after co-culturing with CAR-NK cells, and obtained fluorescence quantification results. The snap point was recorded at 0^th^ and 8^th^ hour. **(B, C)** We also present a diagram **(B)** and micrograph **(C)** for a comparison of cell therapies testing using traditional organoids embedded in BME or PSOs in BME-free conditions. **(D)** We analyzed the mRNA expression level of NKG2D in CAR-NK cells when co-cultured with PSOs or traditional organoids. **(E, F)** We also evaluated the secretion level of IFN-γ **(E)** and perforin **(F)** from CAR-NK cells when co-cultured with PSOs or traditional organoids. Abbreviations used in this study include BF (bright field), FLUOR (fluorescent staining), TO (traditional organoid). “*”, statistically significant difference with the value of p < 0.05. “**”, p < 0.01.

In order to assess the ability of PSOs to screen immunocyte therapies, they were co-cultured with immunocytes such as CAR-NK cells. As seen in **Figure 5A**, the fluorescent live cells staining data showed that PSOs co-cultured with CAR-NK cells quenched faster than those in medium as a control. Through quantification, it was observed that PSOs co-cultured with CAR-NK cells showed a fluorescence reduction from 100% to 13%, whereas in medium, their fluorescence only reduced from 100% to 66% (**Figure 5A**).

The traditional organoid model had several disadvantages when it came to checking immunocyte efficiency through co-culturing methods as immunocytes were unable to directly contact the BME-embedded organoid (as shown in **Figure 5B**). **Figure 5C** represented the “live-action” image of **Figure 5B**. In the left panel, the traditional organoid could not be measured as it was embedded in or under the BME (visible as a shadow block out of focus) and could not be brought to the same plane as the CAR-NK cells (visible as spheroidal cells in focus). However, the PSO was capable of being measured because it was free from BME (visible as a clear block in focus) and in direct contact with CAR-NK cells (visible as spheroidal cells in focus). In order to investigate whether CAR-NK cells could be activated by PSOs or BME-embedded traditional organoids, NKG2D mRNA expression levels of CAR-NK cells were measured. The PSOs and traditional organoids used in the study originated from the same ovarian tumor sample with MUC1 and CD276 positive expression. As shown in **Figure 5D**, both CD276 targeting CAR-NK (CD276-NK) and MUC1 targeting CAR-NK (MUC1-NK) had higher NKG2D expression at 10.1 and 7.9 times, respectively, when they were co-cultured with PSOs as compared to their culturing alone. However, when they were co-cultured with traditional organoids, both CAR-NK cells had lower NKG2D expression at 3.4 and 0.3 times, respectively. In addition to this, CD276-NK and MUC1-NK cells were found to secrete more IFN-γ and perforin when co-cultured with PSOs as compared to traditional organoids, as shown in **Figures 5E, F**.

## Discussion

Our goal was to develop a new platform that could assist cancer patients in selecting individually appropriate chemotherapies or immunocyte therapies for their own tumor samples. Unlike traditional organoids that proliferate from digested cell clusters, PSOs were derived from patients’ primary tumor tissues, which had dissolved mesenchyme, including fibrous tissue and hyaluronic acid. PSOs were more like tiny tumor tissues that were kept alive and separated from the clinical tumor as opposed to an organoid regenerated from a single or several tumor cells. Consequently, the preparation period for PSOs was much shorter than that of traditional organoids, and their characteristics more closely resembled the according tumor tissue.

The preparation process for dissolving mesenchyme from the tumor tissue aimed to allow the primary tumor cells to be soaked in the medium directly and sufficiently without micro-vessel nourishing. However, despite this, we observed that more live tumor cells were gathered at the edge of PSOs with green fluorescence, as shown in **Figure 1B**. Given that PSOs maintained the characteristics of the tumor tissue, they possessed the ability to regenerate primary tumor cells or traditional organoids when digested into single cells or small cell clusters, as demonstrated in **Figure 1C**.

The initial study conducted by *Hagemann, J., et al.* employed patient-derived primary tumor cells to establish spheroids of head and neck squamous cell carcinoma for drug efficacy assessment. By enzymatically digesting biopsy tissues, they obtained single primary cells and then formed spheroids containing approximately 5000 cells per well (26). In a similar vein, the subsequent research by *Hofmann, S., et al.* aimed at generating breast cancer spheroids using 1000 primary tumor cells per well sourced from patients (27). Both promising studies employed the three-dimensional culture method with suspension culture, allowing for convenient drug screening and exploration.

Our approach aligns with the objectives of these investigations, as we successfully developed PSO from gynecological tumor tissues. However, we refined the method by directly culturing minced tissues instead of dissociating them into single cells. Our technique specifically involved the dissolution of intercellular substances while preserving cell-to-cell adhesion and overall tissue structure. This approach offers significant advantages, as it retains the original tumor architecture for further analysis and minimizes the time required, which is particularly valuable for patients, as the regeneration process is bypassed given that the PSO itself represents a viable small tissue structure.

In addition, the gelatin utilized in our study served a similar purpose to that described in the research by *Mazzocchi, A.R.*, providing an adhesive platform for the growth of PSOs or tumor organoids (28).

To ensure that the characteristics of PSOs were consistent with those of tumor tissues, we used one tumor tissue and separated it into two parts for identical detections. One part was immediately prepared for detections such as being digested into single cells for flow cytometry, harvesting mRNA for qRT-PCR measurement, or fixed via paraformaldehyde for IHC staining. The other part was treated per the method described in the “Methods” section to generate PSOs, which were collected the following day for detections similar to their corresponding tumor tissue part. Consequently, we noticed that both PSOs and their corresponding tumor tissues displayed alike structures and the same expression pattern of cellular surface proteins, as demonstrated in **Figures 2A, B**. Moreover, we also used TUNEL staining (chemical development) and the IHC examination of RIPK3 to investigate the necrotic core of PSO. Based on our findings, when the intercellular substance was dissolved thoroughly, the cells located in the core of the PSOs demonstrated survival and were able to access sufficient nutrients. Conversely, in cases where the intercellular substance was not adequately dissolved, PSOs exhibited necrotic cores in the continual culture (**Figure S3**).

To assess whether the viability of PSOs could reflect drug efficacy, we utilized a Live/Dead cell double staining kit to determine the proportions of live and dead cells in PSOs subjected to different concentrations of drug treatments. As seen in **Figures 3A, B**, PSOs showed a gradient change in the proportions of live and dead cells in response to varying concentrations of paclitaxel. Our approach entails using PSOs to mimic clinical methodologies and provide personalized treatment suggestions to patients and doctors. To achieve this, we subject the PSOs to relevant clinical doses of regents and extend the observation period to 5-7 days, as per our current plan, in order to ascertain discernable differences. However, since PSOs from different patients exhibit varied characteristics, some may show insensitivity to certain regents, as depicted in **Figure 4A**. To determine the gradient activity of PSOs when treated with a regent for future drug selection, we employed an excessively high dose of the regent in one group to ensure that the PSOs were completely eliminated. By using this high dose regent, we observed the gradient activity of PSOs at the 48-hour mark after treatment, as illustrated in **Figure 3A**. Surprisingly, during this experiment, we discovered that this particular PSO exhibited unexpected resistance to Paclitaxel when administered at high doses for acute treatment (**Figure 3C**). This finding necessitates further investigation in our future studies. Furthermore, it is worth noting that this phenomenon may have clinical implications, resembling situations where low-dose continuous treatment proves more effective than high-dose acute treatment (29).

Subsequently, we treated PSOs and traditional organoids simultaneously with various drugs. These experiments showed that PSOs exhibited more significant resistance to paclitaxel and oxaliplatin as compared to traditional organoids, as demonstrated in **Figure 3D**. These outcomes suggested that PSOs might maintain resistance to some drugs similar to their corresponding tumor tissues, which played another significant role in pre-clinical drug screening.

We treated PSOs from multiple clinical ovarian cancer samples with several different chemotherapies, including paclitaxel, capecitabine, sirolimus and oxaliplatin, to mimic pre-clinical therapy selecting. As illustrated in **Figure 4A**, the results revealed that different chemotherapies had varying efficacy for the same sample, and different samples showed diverse sensitivities to the same chemotherapies. This emphasized the significance of using PSOs for pre-clinical drug screening for individual patients. To control for variations and minimize errors in our PSO experiments, we employed the strategy of setting parallel wells, as detailed in the Method section. Through technical enhancements, we were able to significantly increase the viability of the PSOs, allowing us to set up 8 parallel wells within each treatment group. As a result, every individual PSO could be analyzed and characterized, with the majority of their features (including drug sensitivities) reflecting those of the corresponding tumor. Moreover, we also planned to improve our method in further compressing the screening time to 72-96 hours, which could be another way for saving patients’ time.

To further investigate whether PSOs could maintain their properties when being passed and regenerated to traditional organoids, we detected the expression of multiple gynecological oncology relevant genes in tumor tissues and followed by PSOs, traditional organoids in P6, and P12. The outcomes indicated that not all genes could maintain their expression properties after multiple passages, which was consistent with the observations presented in the study by Edgar R D, et al., 2022 (20). For instance, in ovarian cancer, some genes’ expression levels such as CEA, FRα, and HE4 could be maintained, while others such as CA125 and HER2 seemed like downregulated for some unknown reason over the passages and regenerations, as illustrated in **Figure 4B**. In addition, we found that OC-02 displayed higher drug resistance, as well as higher expression levels of CA125 and FRα, as compared to other samples. This prompted us to investigate the relationship between CA125 and drug resistance, thus leading to the creation of the CA125 overexpression cell line SKOV-3/CA125+. Using this cell line, we discovered that SKOV-3/CA125+ was more resistant to oxaliplatin, which was consistent with the conclusions reached in the study by Boivin M, et al., 2009 (30), as demonstrated in **Figure 4C**. Furthermore, SKOV-3/CA125+ exhibited greater abilities in terms of migration and regeneration than the normal SKOV-3 cell line, as consistent with the study by Huo Q, et al., 2021 (31), as depicted in **Figure 4D**. These results provided partial justification as to why PSOs exhibit greater resistance to certain chemotherapies than traditional organoids. We have planned to expand our sample collection in order to conduct a more comprehensive transcriptome analysis in our subsequent study. Here, we observed certain inconsistencies in the expression patterns between traditional organoids and PSOs, when we focused on a selection of representative genes associated with gynecological cancer. It suggested that certain features of the primary tumor may carry a risk of being altered during the passaging and culturing period.

Furthermore, with the increasing number of cell therapies being applied in clinical settings, we sought to investigate whether PSO could be suitable for screening in this field. The distinct advantage of PSO is its ability to culture in a BME-Free condition, which enables it to maintain its 3D structure and anchor itself at the bottom of the plate well. This unique property facilitates direct contact between CAR-T cells or CAR-NK cells and PSO (as shown in **Figures 5A–C**), which prompted us to investigate the activation potential of CAR-NK cells when co-cultured with PSO. Interestingly, while traditional organoids derived from the same MUC1 and CD276 positive ovarian tumor tissue showed little or no activation, PSO demonstrated a significant level of activation (as depicted in **Figures 5D–F**).

In summary, we were successful in generating a patient-specific organoid, named PSO, from the tumor tissue of a gynecological cancer patient with a surprisingly short generation period (just one day). This PSO maintained the characteristics of tumor tissues including its structures, relevant gene expression patterns, and resistance to some chemotherapies. Thus, PSO holds great potential for mimicking a patient’s tumor for personalized pre-clinical drug screening. Moreover, it is also a potent tool for cell therapies screening in clinical settings, paving the way for the development of personalized precision medicine.

## Data availability statement

The original contributions presented in the study are included in the article/**Supplementary Material**. Further inquiries can be directed to the corresponding authors.

## Ethics statement

The requirement of ethical approval was waived by Ethics Committee of Cancer Hospital of Shantou University Medical College for the studies involving humans because Ethics Committee of Cancer Hospital of Shantou University Medical College. The studies were conducted in accordance with the local legislation and institutional requirements. The participants provided their written informed consent to participate in this study.

## Author contributions

YZ and ZD designed the experiment, analyzed the data, and wrote the manuscript. ZD, YW, QW, DC and LW collected clinical samples and generated the PSOs and traditional organoids. LW, YuL, XW and YL finished drug screening and relevant measurements. YY, JH and YaL cultured cell lines and performed relevant experiments. HZ and CL proposed the concept, supervised the study and revised the manuscript. All authors contributed to the article and approved the submitted version.

## Supplementary material

Supplementary Figure 1 Fibroblast and tumor cell relevant markers expression pattern. **(A)** CD90, CD105, CD73 and Ki67 expression pattern of the cells migrated out of PSO main body during the 7 days, detected by flow cytometry. **(B)** Ki67 expression pattern of the PSO main body, detected by immunohistochemistry.

Supplementary Figure 2 Wound healing assay of HO8910 and primary cells. **(A)** HO8910 with or without CA125 overexpression, and recorded the snap point at the 0^th^, 11^th^, and 24^th^ hour. **(B)** Primary cells with or without treatment with recombinant human CA125 protein, and recorded the snap point at the 0^th^ and 24^th^ hour.

Supplementary Figure 3 Necrotic core investigation. **(A)** TUNEL staining of PSO with or without thorough dissolving. **(B)** RIPK3 expression in the necrotic core of unsuccessfully dissolved PSO, detected by immunohistochemistry.

Supplementary Table 1 Primers used in the experiments.

Supplementary Table 2 Relevant clinical information of the patients.
