# Supplementary material for: BME-free primary patient-specific organoids obtained with a one-day mimicking method to replicate the corresponding tumor for personalized treatment options
Source: Front Oncol. 2023 Dec 15;13:1239957. doi: 10.3389/fonc.2023.1239957 (PMC10757363; doi:10.3389/fonc.2023.1239957)
Supplement: Supplementary Figure 1 — Fibroblast and tumor cell relevant markers expression pattern. (A) CD90, CD105, CD73 and Ki67 expression pattern of the cells migrated out of PSO main body during the 7 days, detected by flow cytometry. (B) Ki67 expression pattern of the PSO main body, detected by immunohistochemistry. [file DataSheet_1.docx]

**BME-Free Primary Patient-Specific Organoids Obtained with a One-Day** **Mimicking Method to Replicate the Corresponding Tumor for Personalized Treatment Options**

**Supplementary tables**

**Table Legend**

**Table S1.**

Primers used in the experiments.

**Table S2.**

Relevant clinical information of the patients

**Table S1**

| MSLN Forward: | TGGCACAGAAGAATGTCAAG |
| --- | --- |
| MSLN Reverse: | GAATAGCAGCAGGTCCAATG |
| MUC1 Forward: | ATGCTGCTGCTGCTACACTACTT |
| MUC1 Reverse: | TGACTTCTTGACGGTGGTCTTTT |
| CD276 Forward: | TCTCCAAAGGATGCGATAC |
| CD276 Reverse: | GGGTGGTCTGTTCATTGTG |
| ER Forward: | GCAGCACATTAGAGAAAGCCG |
| ER Reverse: | CGGCGGGCCACTTTACTTG |
| CEA Forward: | GATTGGAGTGCTGGTTGGGG |
| CEA Reverse: | TGTTGCAAATGCAGTCTTCCTG |
| FRα Forward: | AGACTGAAAACCCTCTTGAATGC |
| FRα Reverse: | GTCCTCACTGAGTTGGCAACA |
| CA125 Forward: | GTGGCCAAGACAACAACCAC |
| CA125 Reverse: | CGACGGTTATAACTGCTGGTG |
| HE4 Forward: | ATAGCACCATGCCTGCTTGT |
| HE4 Reverse: | TGCTCCTGTGCCTGAGACTA |
| HER2 Forward: | TGCAGGGAAACCTGGAACTC |
| HER2 Reverse: | ACAGGGGTGGTATTGTTCAGC |
| P53 Forward: | TGGATCCGTCTTTCGCGTTTA |
| P53 Reverse: | GGCGGGCCACTTTACTTG |

**Table S2**

| **Patient number** | **Age** | **Main disease** | **Concomitant diseases** | **Menopause** |
| --- | --- | --- | --- | --- |
| OC-01 | 21 | Ovarian carcinoma | Anemia | Pre-menopause |
| OC-02 | 44 | Ovarian granulosa cell tumor | Premature ovarian failure | Post-menopause |
| OC-03 | 43 | Ovarian carcinoma | None | Pre-menopause |
| OC-04 | 34 | Ovarian carcinoma | Hypothyroidism with treatment | Pre-menopause |
| OC-05 | 52 | High-grade ovarian serous cancer | Arrhythmia; Gallstone; Chronic gastritis; Pyloric ulcer; Colonic polyp | Post-menopause |
| CC-01 | 51 | Cervical adenocarcinoma | None | Post-menopause |
| CC-02 | 39 | Cervical squamous cell carcinoma | Bilateral tubal ligation | Pre-menopause |
| CC-03 | 59 | Cervical squamous cell carcinoma | Squamous vaginal intraepithelial neoplasia; Emphysema; Valvular heart disease; Left renal dysplasia; Lumbar disc herniation | Post-menopause |
| CC-04 | 53 | Cervical squamous cell carcinoma | Squamous cell carcinoma of the labia; Hydrohystera; Uterine leiomyoma; Bilateral tubal ligation; Hemorrhoids (after surgery) | Post-menopause |
| CC-05 | 45 | Cervical squamous cell carcinoma | Uterine leiomyoma; Pelvic endometriosis; Hypertension | Pre-menopause |
| EC-01 | 56 | 1. Endometrial adenocarcinoma | 1. Ovarian mucinous cystadenoma; Diabetes | Pre-menopause |
| EC-02 | 64 | 1. Endometrial adenocarcinoma | 1. Hypertension; Hepatic cyst; Cervical polyp | Post-menopause |
| EC-03 | 72 | 1. Endometrial adenocarcinoma | 1. None | Post-menopause |
| EC-04 | 51 | 1. Endometrial adenocarcinoma | 1. None | Pre-menopause |
| EC-05 | 63 | 1. Endometrial serous carcinoma | 1. Bilateral hydrosalpinx; Hydrohystera; Diabetes; Hypertension | Post-menopause |
| EC-06 | 61 | 1. Endometrial adenocarcinoma | 1. Uterine leiomyoma; Diabetes; Hypertension; Cerebral infarction (after surgery); Fatty liver; Right renal hamartoma; Hyperuricemia | Post-menopause |

**Figure S1**

**
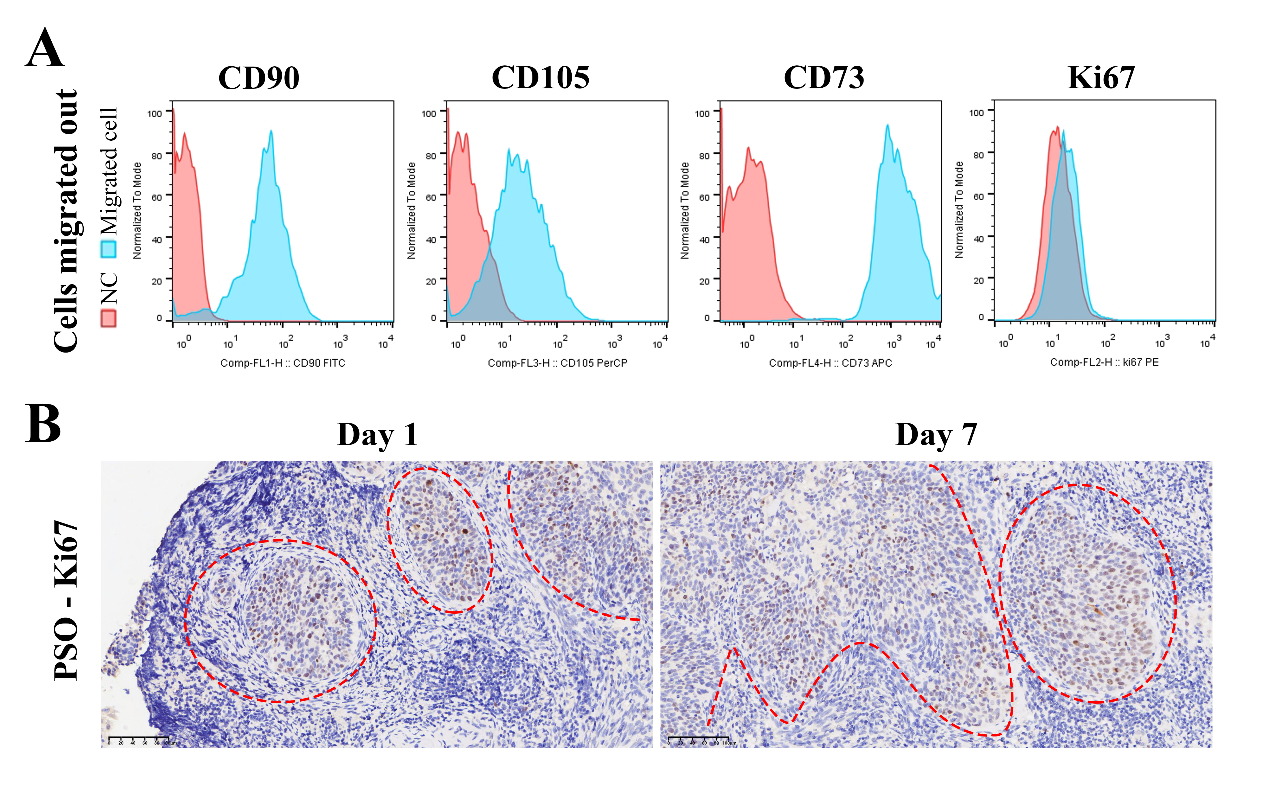
**

**Figure S2**

**
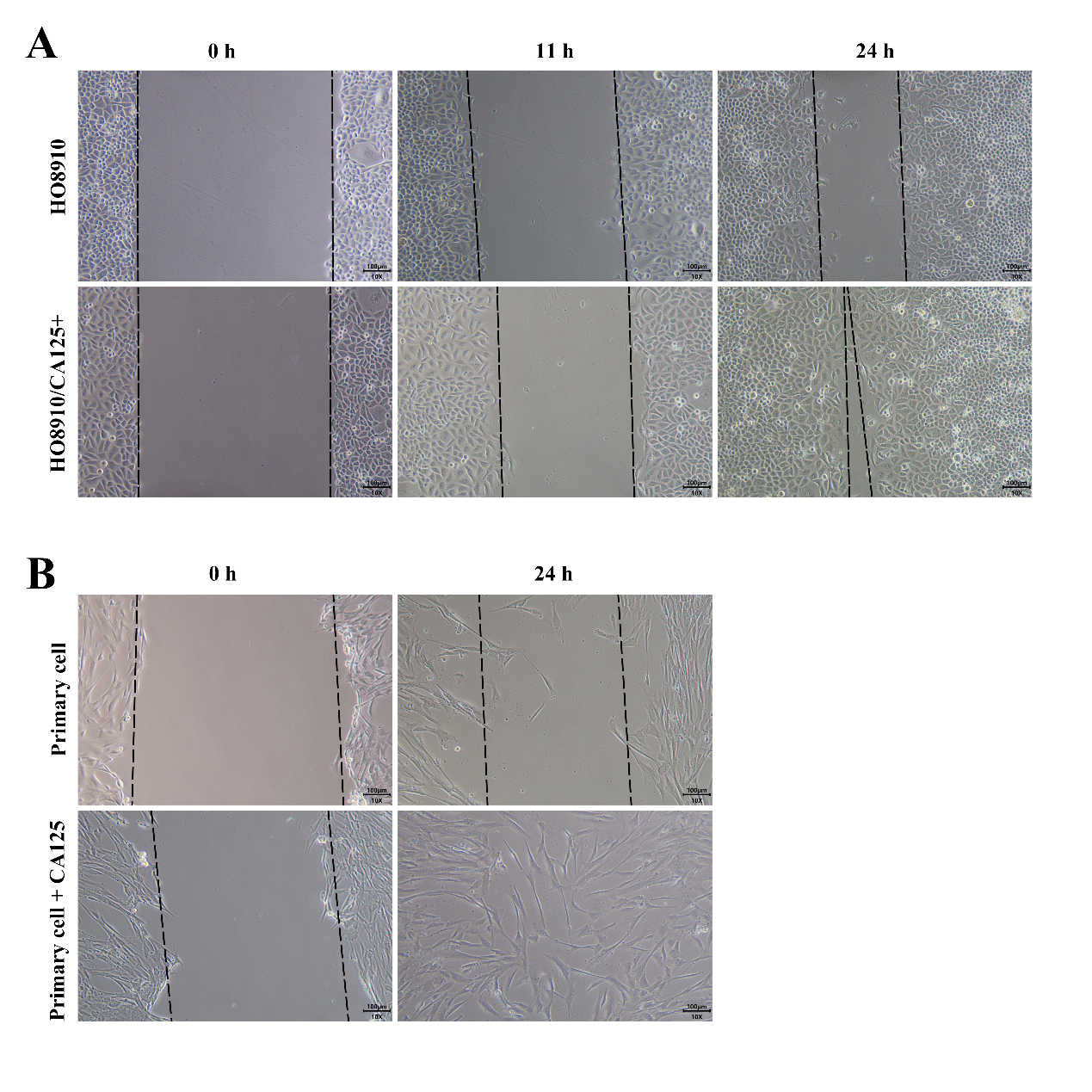
**

**Figure S3**

**
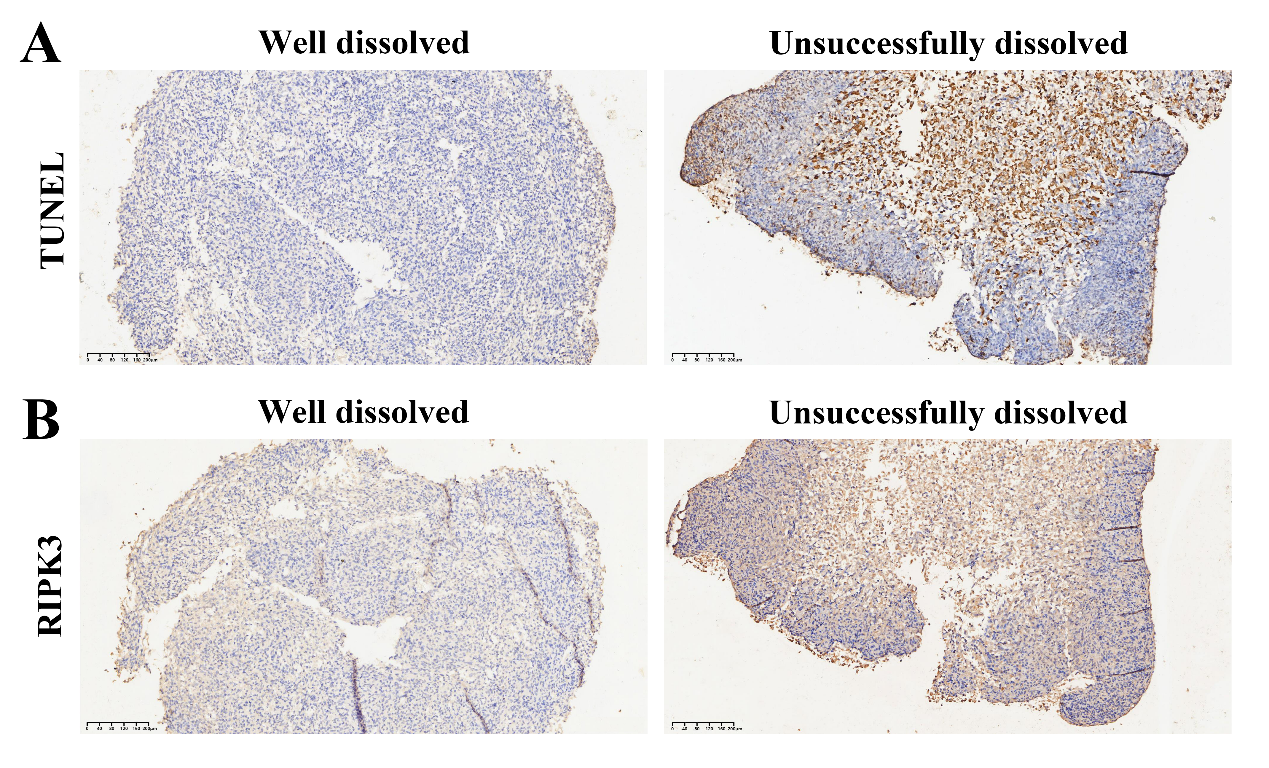
**
